# Exploring the relationships among art therapy students’ burnout, practicum stress, and teacher support

**DOI:** 10.3389/fpsyg.2023.1230136

**Published:** 2023-08-03

**Authors:** Juliet Jue, Tae-Eun Kim

**Affiliations:** ^1^Department of Art Therapy, Hanyang Cyber University, Seoul, Republic of Korea; ^2^Graduate School of Art Therapy, CHA University, Pocheon, Republic of Korea

**Keywords:** teacher support, art therapy students, burnout, practicum stress, academic support

## Abstract

**Introduction:**

This study examines how teacher support and practicum stress affect art therapy graduate students’ burnout.

**Methods:**

A total of 125 master’s and doctoral students from art therapy graduate schools in Korea participated in the study. We conducted a correlation analysis and multiple regression analysis to explore the relationship between the variables.

**Results:**

The correlation analysis results showed that burnout, practicum stress, and social support are significantly interrelated. The regression analysis results indicated that practicum stress increases burnout while social support decreases it. When we used the sub-factors of social support as independent variables, we found that professor support rather than the support of colleagues or family significantly reduced burnout. When we divided the perceived teacher support into emotional support and academic support, our analysis identified that academic support was more important than emotional support to reduce students’ burnout.

**Conclusion:**

Art therapy students’ practicum stress can cause psychological burnout, while teacher support—especially academic support—can lower the possibility of experiencing such burnout.

## 1. Introduction

Graduate students who enroll in art therapy graduate school complete practicum while taking classes. Practicum is important because it provides students opportunities to meet clients in the real world. Engaging with clients in art therapy sessions is the cornerstone of learning art therapy. However, unsurprisingly, practicum is sometimes very challenging and even stressful. Despite receiving help from field and school supervisors, students often encounter obstacles over the course of their practice, including difficulties in understanding their clients, lack of therapeutic progress, the emotional burden of the therapist-patient relationship, unexpected termination of sessions, feeling disrespected by other staff, administrative problems/legal issues, and work overload. Simply put, art therapy graduate students may find practice quite stressful, and exhausted students sometimes withdraw from school temporarily or drop out.

The term “burnout” refers to a type of physical and mental exhaustion featuring loss of motivation and cynical attitudes that frequently occurs in service professionals (Raquepaw and Miller, 1989; Skovholt and Trotter-Mathison, 2016). Maslach and Jackson (1981) viewed burnout as a syndrome whose main symptoms among helping professionals included emotional exhaustion, depersonalization, and lack of personal accomplishments. When therapists suffer burnout, they experience fatigue, lethargy, and a loss of energy, ideals, and sense of purpose in their work. The resulting loss of motivation lowers job performance, and decreases positive feelings for or interest in clients. As a result, the quality of the services deteriorates, and the therapists themselves lose self-confidence and self-esteem.

Graduate students training to become professional therapists are no exception to the risk of burnout (Choi, 2013). Job stress, or practicum stress in case of students, is one of the risk factors for worsening overall mental states. Unfortunately, some art therapy students experience overwork, lack of resources, excessive demands and/or conflicts with the authorities during practicum. In such work environments, the likelihood of experiencing burnout increases (Choi et al., 2002; Lee et al., 2011; Wallace et al., 2011). The more the student therapists experience stress at work, the more likely they are to experience burnout. Previous studies have shown that psychotherapists often experience burnout because high stress levels are a distinctive feature of the profession (Raquepaw and Miller, 1989; Ross et al., 1989; Delia and Patrick, 1996; Moore and Cooper, 1996; Yoo and Park, 2002; Park, 2007).

Although risk factors exist, protective factors such as social support can help prevent burnout (Choi and Chung, 2003). Social support can be defined as all the positive resources and experiences an individual can obtain from interpersonal relationships (Taylor, 2011); it can alleviate the negative effects of job stress and reduce burnout (Sánchez-Moreno et al., 2015). Indeed, emotional tension and stress can be reduced if therapists receive social support (Delia and Patrick, 1996; Park and Park, 2018).

Previous studies have found that the influence of social support on burnout varies based on the type of social support therapists receive. For therapists, the sources of social support can be roughly divided into family members, colleagues, and supervisors. Some studies have reported that support from family or colleagues is more effective than supervisors’ support (Yoo and Park, 2002; Choi and Chung, 2003). Others have reported that support from supervisors is more important in lowering burnout levels, especially among beginner therapists (Bang, 2006; Lee et al., 2009; Hyun and Hong, 2018; Lee et al., 2019).

In this context, the term “supervisor” refers to a qualified expert who can provide supervision. In art therapy graduate programs, professors play supervisory roles, helping student supervisees reflect on their inner states, treatment plans, and interventions and make optimal decisions. Overall, supervisors not only help students learn, but also provide their supervisees emotional support (Keum and Son, 2017). Supervisors encourage and empower beginner therapists by providing considerate feedback and constructive advice, helping them build self-confidence (Kim, 2018).

Although researchers have studied burnout among art therapists working in the field (Kim and Jeong, 2012; Kim, 2015; Kim and Kim, 2020), few studies have focused on beginner art therapists in graduate school. To understand the growth of art therapy graduate students as experts and the quality of their practicum, it is necessary to determine how much stress or burnout students experience during their practicum and to what extent protective factors such as social support are helpful and what are needed. This information can be an indicator of whether they will successfully complete their training and become experts. Examining the degree of psychological exhaustion experienced by art therapy graduate students and what factors increase or decrease their burnout is critical. In this study, we set out to examine how much teacher support can reduce student’s burnout caused by practicum stress, and to verify what kind of teacher support is most beneficent.

## 2. Materials and methods

### 2.1. Participants

The participants were Korean art therapy graduate students. Art therapy was first introduced in Korea in the late twentieth century, followed by the establishment of the Korean Art Therapy Association in 1991 (Kim, 2009; Choi, 2013). In a decade, more than 10 art therapy graduate schools were established in Korea (Kim, 2009), and as of 2023, there are more than 30 art therapy graduate schools. Most graduate schools offer master’s degree programs, and some offer doctoral degrees. All graduate schools operate on a semester system, and art therapy graduate students generally spend two to 3 years (four to six semesters) in their master’s programs. Although practicum is not mandatory for graduation, they must take an art therapy practicum to obtain a national license after graduation. The curriculum of most Korean art therapy graduate schools includes supervision as one of the classes, and students enrolled in practicum attend supervision classes.

A total of 125 art therapy graduate students in Korea—81 master’s degree students and 44 doctoral students—participated in this study. We recruited students who had completed two or more semesters. The master’s students had been enrolled for an average of 3.8 semesters (S.D. = 1.0, Min., 2, Max., 6), and the doctoral students had been enrolled for an average of 3.5 semesters (S.D. = 1.3, Min., 2, Max., 5). Participants’ ages ranged from 25 to 60 years, and the mean age was 39.5 years old (S.D. = 8.6 years). The gender distribution ratio was five males (4.0%) and 120 females (96.0%). The high percentage of females reflects the gender ratio of all graduate students in art therapy schools; the ratio is similar to that of Korean art therapists reported at a recent conference (Lee et al., 2017).

In Korea, the gender ratio of art therapists skews toward females. For example, Kim and Jeong (2012) collected data from 107 art therapists, and their gender ratio was 104 females (97.2%) and 3 males (2.8%). Another study with 128 Korean art therapists showed the participants’ gender ratio was 120 females (93.75%) and 8 males (6.25%) (Kim and Kim, 2020). The low proportion of male participants was also consistent in qualitative studies. In addition, Choi’s (2013) qualitative research on art therapy graduate students included in-depth interviews with 16 female students from three graduate schools.

### 2.2. Measures

#### 2.2.1. The Maslach burnout inventory

Maslach and Jackson (1981) developed the Maslach Burnout Inventory (MBI), and Park (2001) translated it into Korean and validated it. MBI is composed of 22 items, assessing counselors’ burnout. It has three sub-scales: emotional exhaustion (9 items), depersonalization (5 items), and personal accomplishment (8 items). It uses a 7-point Likert scale (0 = Not at all, 6 = everyday). The personal accomplishment items are summed using inverse scoring, and the other items are summed as they are. The total score ranges from 0 to 132. The higher the final score, the greater the degree of burnout. Internal consistencies, measured by Zumbo ordinal αs, were as follows: emotional exhaustion, α = 0.91; depersonalization, α = 0.71; and personal accomplishment, *α* = 0.91.

#### 2.2.2. The workplace stress scale

To examine art therapy students’ practicum stress, we used the workplace stress scale, originally developed by Jayaratne and Chess (1983) and later translated into Korean and validated by Yoon (2000). This scale has 19 items, comprising four sub-variables: challenge (6 items), role conflict (5 items), role ambiguity (3 items), and work overload (5 items). Challenge evaluates work autonomy and opportunities to develop competency. Role conflict assesses the degree of conflict experienced when job performance demands are inconsistent with or contradictory to personal standards. Role ambiguity measures the uncertainty of role performance as a condition in which individuals are not sufficiently informed about how to perform their roles. Work overload evaluates whether a given amount of work exceeds an individual’s time and capacity. This scale uses a 5-point Likert scale (1: not at all, 5: highly agree), and the positively described items are reverse-scored and summed. The final score ranges from 19 to 95, and a higher score means a higher level of stress in the workplace. The Zumbo ordinal αs for this scale were as follows: challenge, α = 0.77; role conflict, α = 0.73; role ambiguity, α = 0.89; and work overload, *α* = 0.83.

#### 2.2.3. The social support scale

The Social Support Scale was originally developed by Caplan et al. (1980), and Park (2001) translated it into Korean. It measures the level of social support counselors perceive themselves as receiving from supervisors, peers, and family members. In Korean art therapy graduate schools, professors served as supervisors. To avoid any confusion with a field supervisor, we replaced the term “supervisor” with “professor” in this study. This scale uses a 5-point Likert scale (1 point: not at all, 5 points: highly agree), and each category includes 6 items. The score for each category ranges from 6 to 30 points, and the highest total score for the 3 categories is 90 points. A higher score means more social support they received. The reliability scores measured by Zumbo ordinal *α* for this scale was 0.90 in this study.

#### 2.2.4. The teacher support scale

To measure students’ perceived teacher support in detail, we used the Teacher Support Scale developed by Seok (2007) based on Hektner (1996) and later modified by Kim (2019). This scale consists of 10 total items divided into two sub-factors: academic support (five items) and emotional support (five items). The former measures the degree to which teachers provide academic support to help students cope with problems in class, provide appropriate feedback to questions, and help students improve knowledge and skills in the field. The latter evaluates the extent to which teachers listen courteously and encourage students. This scale uses a 5-point Likert scale, and a higher score means a higher level of support from professors. We found the Zumbo ordinal α as follows: academic support, *α* = 0.89; and emotional support, *α* = 0.86.

### 2.3. Procedures and ethical consideration

Before starting the survey, we received approval from the Institutional Review Board of the researcher’s institution. We contacted five art therapy graduate schools and posted flyers asking art therapy students to participate in the study. We ensured that the study participants understood that answering the questionnaire was completely voluntary, that their anonymity was guaranteed, and that data would be destroyed after the study was completed. They responded to the questionnaire after signing a consent form.

### 2.4. Analysis method

To test our hypothesis, we calculated descriptive statistics and conducted a correlation analysis among the variables. We then used multiple regression analysis to explore the relationship between the variables.

## 3. Results

### 3.1. Descriptive statistics and correlation analysis

To understand the overall relationship among variables, we calculated descriptive statistics and conducted a correlation analysis. Table 1 presents the results. The total scores for practicum stress and burnout were positively correlated (*r* = 0.45, *p* < 0.001). In detail, all of the sub-variables of practicum stress except role conflict (*r* = 0.16, *n.s*.) showed significant correlation results with burnout. The significant three sub-factors include challenge (*r* = 0.47, *p* < 0.001), role ambiguity (*r* = 0.27, *p* < 0.01), and work overload (*r* = 0.22, *p* < 0.05).

**Table 1:** Correlation coefficients and descriptive statistics for measurement variables.

	M	SD	1	2	3	4	5
**1. Practicum stress**	46.89	8.22	–				
2. Challenge	13.74	3.49	0.66^***^	–			
3. Role conflict	12.80	3.41	0.78^***^	0.30^**^	–		
4. Role ambiguity	6.45	2.07	0.39^***^	0.35^***^	0.12	–	
5. Work overload	13.85	3.86	0.64^***^	0.06	0.45^***^	−0.12	–
**6. Social Support**	69.47	10.81	−0.26^**^	−0.35^***^	−0.02	−0.27^**^	−0.04
7. Professor support	22.20	4.91	−0.44^***^	−0.53^***^	−0.14	−0.25^**^	−0.18^*^
8. Peer support	22.98	5.10	−0.23^*^	−0.24^**^	−0.08	−0.11	−0.11
9. Family support	24.30	4.72	0.10	−0.01	0.18^*^	−0.23^**^	0.22^*^
**10. Teacher support**	40.15	7.22	−0.35^***^	−0.47^***^	0.09	−0.33^***^	−0.08
11. Academic support	20.57	3.61	−0.38^***^	−0.49^***^	0.12	−0.34^***^	−0.09
12. Emotional support	19.58	3.92	−0.30^**^	−0.41^***^	−0.07	−0.29^**^	−0.07
**13. Burnout**	37.06	14.57	0.45^***^	0.47^***^	0.16	0.27^**^	0.22^*^

	6	7	8	9	10	11	12	13
**1. Practicum stress**								
2. Challenge								
3. Role conflict								
4. Role ambiguity								
5. Work overload								
**6. Social Support**	–							
7. Professor support	0.70^***^	–						
8. Peer support	0.85^***^	0.47^***^	–					
9. Family support	0.64^***^	0.06	0.38^***^	–				
**10. Teacher support**	0.67^***^	0.86^***^	0.46^***^	0.14	–			
11. Academic support	0.62^***^	0.80^***^	0.41^***^	0.15	0.96^***^	–		
12. Emotional support	0.66^***^	0.85^***^	0.47^***^	0.13	0.96^***^	0.84^***^	–	
**13. Burnout**	−0.36^***^	−0.45^***^	−0.26^**^	−0.09	−0.38^***^	−0.38^***^	−0.35^***^	–

^*^ *p* < 0.05, ^**^ *p* < 0.01, ^***^ *p* < 0.001.

Next, our analysis revealed a negative correlation between social support and burnout (*r* = −0.36, *p* < 0.001). Regarding the sub-variables of social support in detail, we found that professor support (*r* = −0.45, *p* < 0.001) and peer support (*r* = −0.26, *p* < 0.01) were significant in their negative correlations with burnout, but family support (*r* = 0.09, *n.s.*) was not significantly correlated with burnout.

The total value of teacher support was negatively correlated with burnout (*r* = −0.38, *p* < 0.001). Both sub-variables, academic support (*r* = −0.38, *p* < 0. 001) and emotional support (*r* = −0.35, *p* < 0. 001), showed negative correlations with burnout.

### 3.2. Multiple regression analysis

To verify relative influence of each variable, we conducted multiple regression analyzes and their results are presented in Table 2. A Durbin-Watson test score of 1.7, close to the value of 2, confirmed that there was no autocorrelation. Meanwhile, the Tolerance and the Variance Inflation Factor (VIF) values indicated no multicollinearity. Finally, the enter method-based multiple regression analysis verified that the model was valid and that each path was significant—practicum stress (*p* < 0.001) and social support (*p* < 0.01). The non-standardized coefficient of practicum stress (B = 0.68) was positive, while that of social support (B = −0.37) was negative. This indicates that practicum stress increases burnout, while perceived social support decreases it. In addition, the effect size of the variables implied that practicum stress had a stronger effect than social support, as the β of practicum stress was 0.38 and that of social support was −0.27. The total explanatory power of the model was 27%.

**Table 2:** The effect of practicum stress and social support on burnout.

Variable	B	SE	*β*	*t*	*p*-value
Practicum stress	0.68	0.15	0.38	4.57^***^	0.000
Social support	−0.37	0.11	−0.27	−3.29^**^	0.001
*R^2^*	0.27				
*Adj. R^2^*	0.26				
*F*	21.16^***^				
Durbin-Watson	1.68				

^**^ *p* < 0.01, ^***^ *p* < 0.001.

**Figure 1:**
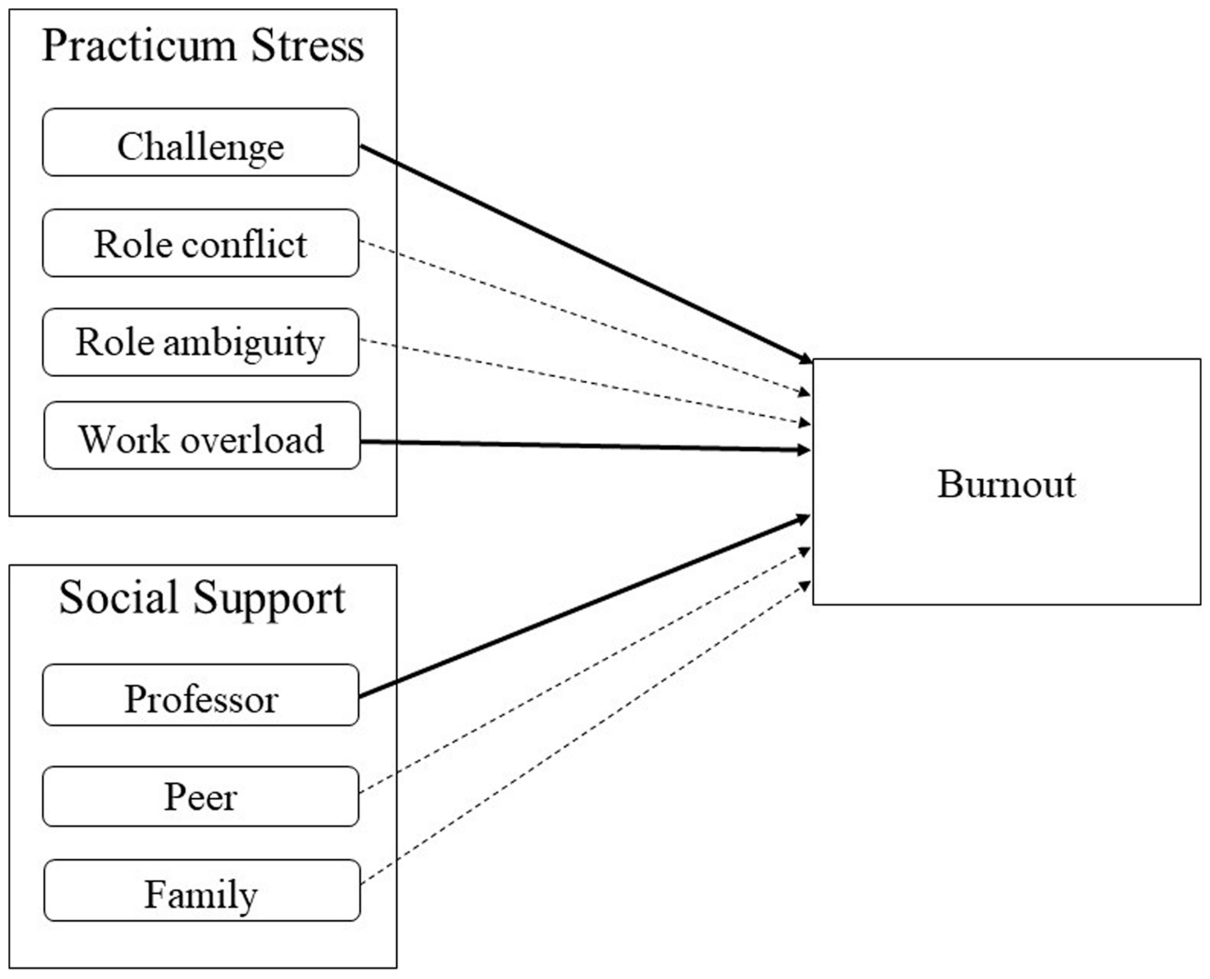
The effect of all practicum stress and social support sub-variables on burnout. Solid lines are significant, while dotted lines are not.

Next, we set sub-variables of social support and those of practicum stress as independent variables, and examined which sub-variable had a significant effect. Table 3 and Figure 1 show the results of this analysis. The Durbin-Watson test score was 1.7, indicating that it was appropriate to use this regression model. We found no multicollinearity; the model’s tolerance was above 0.1 and VIF was less than 10. The significance probability of this model was 0.000, and we verified the significance of each path; challenge (*p* < 0.01), work overload (*p* < 0.05), and professor support (*p* < 0.05) had significant effects on burnout. Although role ambiguity and peer support showed significant correlations with burnout, their relatively weak explanatory power in the regression analysis demonstrated their insignificance. Among the significant variables, the most influential variable was challenge (β = 0.33), followed by professor support (*β* = −0.22), and work overload (*β* = 0.20). Finally, the independent variables’ explanatory power was 34%.

**Table 3:** The effect of all practicum stress and social support sub-variables on burnout.

Variable		B	S.E.	*β*	*t*	*p*-value
Practicum stress	Challenge	1.39	0.42	0.33	3.36^**^	0.001
	Role conflict	−0.23	0.41	−0.05	−0.57	0.57
	Role ambiguity	0.85	0.63	0.12	1.35	0.18
	Work overload	0.76	0.36	0.20	2.13^*^	0.04
Social support	Professor	−0.66	0.31	−0.22	−2.13^*^	0.04
	Peer	−0.08	0.28	−0.03	−0.27	0.78
	Family	−0.15	0.29	−0.05	−0.52	0.61
*R^2^*		0.34				
*Adj. R^2^*		0.30				
*F*		8.12^***^				
Durbin-Watson		1.72				

^*^ *p* < 0.05, ^**^ *p* < 0.01, ^***^ *p* < 0.001.

**Figure 2:**
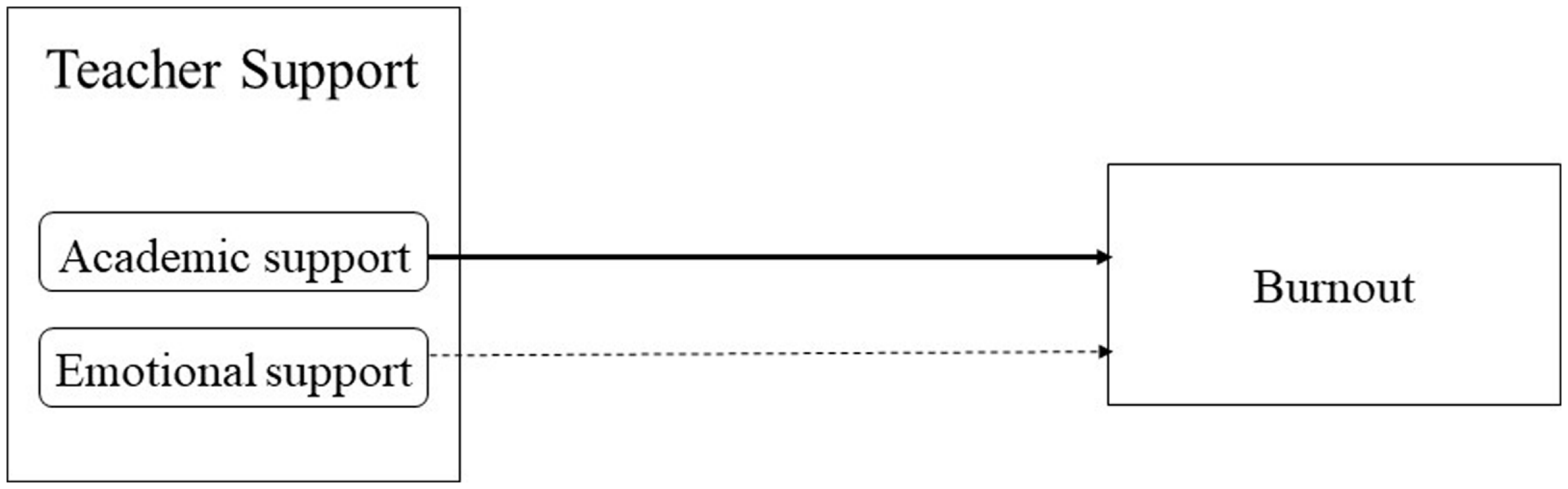
The effect of teacher support sub-variables on burnout. Solid lines are significant, while dotted lines are not.

In this second analysis with the sub-variables, we found that professor support was an important component of social support. Based on the results, we conducted a third regression analysis to determine content of teacher support. We set the two sub-variables of teacher support—academic support and emotional support—as independent variables, and burnout as a dependent variable. We conducted enter method-based multiple regression analysis, and their results are presented in Table 4 and Figure 2. We found the significance probability of this model to be 0.000, and the total explanatory power of the variables was 15%. The regression analysis identified academic support (*p* < 0.001) as the only significant variable, although both emotional and academic support showed significant negative correlations with burnout. These results suggest that the important factor in teacher support to lower students’ burnout is academic support rather than emotional support.

**Table 4:** The effect of teacher support sub-variables on burnout.

Variable	B	S.E.	*β*	*t*	*p*-value
Academic support	−1.54	0.34	−0.38	−4.59^***^	0.00
Emotional support			−0.11	−0.71	0.48
*R^2^*	0.15				
*Adj. R^2^*	0.14				
*F*	21.10^***^				
Durbin-Watson	1.74				

^***^ *p* < 0.001.

## 4. Discussion

We examined the relationship between art therapy students’ burnout, practicum stress, and teacher support in this study. Regarding social support, we focused on assistance from professors. The results and implications of this study are as follows.

First, we found that art therapy students’ practicum stress significantly increases burnout. The analysis of sub-variables showed that the risk of burnout was highest when students lacked autonomy or opportunity for skill development at work. Excessive work was the second cause of burnout. These results are consistent with previous studies showing that work risk factors induce job stress and subsequently increase the potential for burnout (Raquepaw and Miller, 1989; Yoon, 2000; Do and Chung, 2009; Moyer, 2011; Jang and Yu, 2013; Yun, 2013). In particular, our results are consistent with findings indicating that counselors’ psychological tiredness increases when they do not receive sufficient challenges at work or are subjected to excessive workloads (Brewer and Clippard, 2002; Baggerly and Osborn, 2006; Hakanen et al., 2006; Gibson et al., 2009; Yun and Chung, 2011). Psychotherapists belonging to institutions reported less autonomy and lower levels of personal achievement than those in private practice, and the former were more vulnerable to burnout (Rupert and Morgan, 2005). Therefore, it is very important for therapists, whether trainees or professionals, to feel adequate autonomy, control at work, and be motivated while developing their abilities. If this autonomy and motivation are interrupted, the likelihood of burnout increases.

While previous studies have identified role conflict as the most important contributor to job stress (Park and Yoon, 2011; Park and Hang, 2017), it was not associated with burnout among art therapy students in our study. Presumably, this discrepancy is a result of their different professional status. Graduate students are both therapists and trainees who undergo supervision during art therapy practice, so even if their roles are limited, they probably accept these limitations and experience fewer role conflicts.

Second, we found that social support can reduce the possibilities of experiencing burnout. As mentioned in the introduction, social support helps relieve burnout (Cohen and Wills, 1985; Ross et al., 1989; Brown and O’Brien, 1998; Baruch-Feldman et al., 2002). When social support is low, it is hard to deal with stress adaptively, but when it is high, people can use it as a psychological resource to help them get through stressful situations (Choi and Chung, 2003).

Furthermore, our analysis with the sub-factors of social support found that professor support had the most significant impact, while the effects of colleague and family were relatively insignificant. This result should be carefully considered in that participants were graduate students in Asian society where the family is the greatest support for psychological consolation. Researchers who studied the relationship between social support and burnout of Korean professional counselors found that family support and peer support were more important than supervisor support (Park, 2001; Yoo and Park, 2002; Choi and Chung, 2003). Their findings underlined the importance of emotional support from family members in the family-centered culture of Korea. Therefore, our results are contrary to what is expected in light of the specificity of the Asian culture. To understand the contradiction, we paid attention to the differences in study subjects, the occupational status of the subjects. The previous studies’ participants comprised paid professional counselors; our subjects were art therapy graduate students practicing as interns. For professional counselors, emotional intimacy with those around them or recognition and respect from significant others is more important in preventing psychological burnout than obtaining information needed to cope with problems. On the other hand, art therapy graduate students are pre-experts with room for improvement rather than performing independent functions as experts. It is plausible that teacher support could be more important than support from other sources to them, as teachers understand the specific stresses trainees experience and can provide more necessary assistance than others.

Further analysis appears to support this explanation: The examination of the components of teacher support identified academic support as more important than emotional support. It also contradicts the conventional wisdom held by family-oriented societies in Asia that emotional closeness to those around them or approval from important people are more crucial in preventing psychological exhaustion. In other words, for students, obtaining information necessary to understand and cope with various problems encountered in practice and achieving professional growth is indeed the way to overcome burnout. The importance of academic support has also been confirmed in previous studies (Johnston and Milne, 2012; Wilson et al., 2016). Wilson et al. (2016), who analyzed 15 studies on supervision, found that 13 of them considered learning opportunities to be the most important factor in supervision. Emotional support and the teacher-student relationship were key factors, but learning was also found to be a crucial component in overcoming adversity and becoming professionals. Our result also highlights the importance of teachers’ academic support in higher education. Appropriate guidance from teachers can reduce emotional exhaustion, help individuals find meaning in their work, and increase their sense of accomplishment.

Referring to the practicum distress of art therapy students, Van Lith and Voronin (2016) pointed out that “students fluctuate through periods of uncertainty and feelings of being a fraud” (p. 53). Similarly, Rønnestad and Skovholt (2003) stated that students experience “performance anxiety, … a sense of being fragile and incomplete as a practitioner, insufficient conceptual maps, … and a feeling of neediness for mentors” (p. 45) during their practicum. Thus, art therapy graduate students are in need of academic support more desperately than emotional support. Feldstein (2000) also reported that school counselors who received clinical supervision experienced less emotional burnout than those with non-clinical supervision. Therefore, in order to alleviate the psychological burnout of art therapy graduate students in practicum, it is essential to provide academic support to help them improve their expertise.

The implications of this study for art therapy education and practice are as follows. First, the absence of challenge and work overload in the practicum locations might affect students’ burnout. Therefore, preventing burnout requires the creation of practice conditions that can offer trainees appropriate degrees of autonomy, such as letting them design their schedules or processes. In addition, examining student therapists’ workloads and maintaining appropriate workload levels are critical. Second, an interesting finding from this graduate program with a mandatory practicum is the significance of teacher assistance, particularly academic help, in reducing the likelihood of psychological burnout in students. It implies that teachers should consider academic growth and professional development as major things in providing emotional, informational, material, and evaluative support to their students.

The limitations of this study and suggestions for future studies are as follows. First, this study used a quantitative approach to verify the relationship between stress, burnout, and teacher support in art therapy graduate students, but it did not carefully capture the difficulties they experience in practice or the psychological changes they feel when receiving help from professors. In future research, it is necessary to take a qualitative and in-depth approach to examine under what circumstances they experience stress, feel the risk of psychological burnout, and disclose the nature of support that reduces the risk of burnout. Second, we considered a non-comprehensive set of protective factors against burnout, focusing on social support and professor support. Therefore, future studies should endeavor to identify other protective factors and to develop a model for the psychological burnout path. Third, we used a cross-sectional approach, administering a survey to participants at a specific point in time and performing statistical analysis based on the results. Thus, we merely sought to estimate cause and effect; our findings provide no conclusive evidence for a causal relationship. In the future, researchers should seek to clarify the causal relationship by undertaking longitudinal studies. Finally, this study used self-report questionnaires, which means participants may have answered in ways they deemed socially desirable. Future study should consider combining quantitative and qualitative approaches by conducting interviews.

## 5. Conclusion

This study examined the relationship between practicum stress, psychological burnout, and teacher support in art therapy graduate students, a topic that researchers have not previously studied. Psychological burnout develops gradually and greatly impacts professional and personal life quality. We found that practicum stress can cause psychological burnout, while teacher support can lower the possibility of experiencing such burnout. Therefore, this study’s results will help graduate education administrators establish a direction to enable art therapy graduate students to cope with and prevent psychological burnout.

## Data availability statement

The raw data supporting the conclusions of this article will be made available by the authors, without undue reservation.

## Ethics statement

The studies involving human participants were reviewed and approved by Hanyang Cyber University Institutional Review Boards. The patients/participants provided their written informed consent to participate in this study.

## Author contributions

T-EK conceived of the presented idea. T-EK and JJ developed the theory and conducted survey. JJ verified the analytical methods, analyzed the data, and wrote the manuscript in consultation with T-EK. All authors contributed to the article and approved the submitted version.

## Conflict of interest

The authors declare that the research was conducted in the absence of any commercial or financial relationships that could be construed as a potential conflict of interest.

## Publisher’s note

All claims expressed in this article are solely those of the authors and do not necessarily represent those of their affiliated organizations, or those of the publisher, the editors and the reviewers. Any product that may be evaluated in this article, or claim that may be made by its manufacturer, is not guaranteed or endorsed by the publisher.
